# Selective crystallization of indigo B by a modified sublimation method and its redetermined structure

**DOI:** 10.1107/S1600536811040220

**Published:** 2011-10-08

**Authors:** Florian Kettner, Lucie Hüter, Johanna Schäfer, Konstantin Röder, Uta Purgahn, Harald Krautscheid

**Affiliations:** a Universität Leipzig, Fakultät für Chemie und Mineralogie, Institut für Anorganische Chemie, Johannisallee 29, D-04103 Leipzig, Germany; b Spezialschulteil Mathematik/Naturwissenschaften/Informatik am, Albert-Schweitzer-Gymnasium Erfurt, Vilniuser Strasse 17a, D-99089 Erfurt, Germany

## Abstract

Good-quality single crystals of the title compound, indigo B [systematic name: 2-(3-oxoindolin-2-yl­idene)indolin-3-one], C_16_H_10_N_2_O_2_, have been prepared with high selectivity by a sublimation process. The previous structure of indigo B [Süsse & Wolf (1980 ▶). *Naturwissenschaften*, **67**, 453], which showed that the complete mol­ecule is generated by crystallographic inversion symmetry has been confirmed, but the present study reports more realistic geometrical parameters and modern standards of precision (e.g. σ for C—C bonds = 0.002–0.003 Å). Each mol­ecule features two intra­molecular N—H⋯O hydrogen bonds. In the crystal, mol­ecules are linked by strong face-to-face π–π stacking inter­actions involving both the six- and five-membered rings [centroid–centroid separations = 3.6290 (14) and 3.6506 (14) Å] and inter­molecular N—H⋯O hydrogen bonds.

## Related literature

For background to the history and uses of indigo, see: Berger & Sicker (2009 ▶); Johnson-Buck *et al.* (2009 ▶). For previous studies of the polymorphism of indigo, see: von Eller (1955 ▶); von Eller-Pandraud (1958 ▶); Süsse & Wolf (1980 ▶); Süsse *et al.* (1988 ▶). For aromatic stacking, see: Meyer *et al.* (2003 ▶).
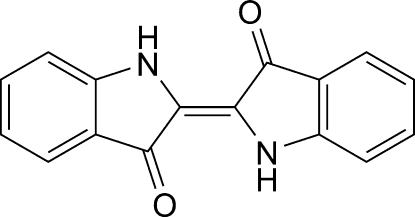


## Experimental

### 

#### Crystal data

C_16_H_10_N_2_O_2_
*M* *_r_* = 262.26
Monoclinic, 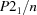

*a* = 9.799 (2) Å
*b* = 5.9064 (10) Å
*c* = 10.755 (3) Å
β = 106.781 (18)°
*V* = 596.0 (2) Å^3^
*Z* = 2
Mo *K*α radiation
μ = 0.10 mm^−1^
*T* = 213 K
1.00 × 0.50 × 0.30 mm

#### Data collection

Stoe IPDS 1 diffractometer
4479 measured reflections
1115 independent reflections
896 reflections with *I* > 2σ(*I*)
*R*_int_ = 0.033

#### Refinement

*R*[*F* ^2^ > 2σ(*F* ^2^)] = 0.036
*wR*(*F* ^2^) = 0.096
*S* = 1.03
1115 reflections
91 parameters
H-atom parameters constrained
Δρ_max_ = 0.20 e Å^−3^
Δρ_min_ = −0.16 e Å^−3^

### 

Data collection: *X-AREA* (Stoe & Cie, 2005 ▶); cell refinement: *X-AREA*; data reduction: *X-AREA*; program(s) used to solve structure: *SHELXS97* (Sheldrick, 2008 ▶); program(s) used to refine structure: *SHELXL97* (Sheldrick, 2008 ▶); molecular graphics: *DIAMOND* (Brandenburg, 2004 ▶); software used to prepare material for publication: *WinGX* (Farrugia, 1999 ▶).

## Supplementary Material

Crystal structure: contains datablock(s) global, I. DOI: 10.1107/S1600536811040220/hb6323sup1.cif

Structure factors: contains datablock(s) I. DOI: 10.1107/S1600536811040220/hb6323Isup2.hkl

Supplementary material file. DOI: 10.1107/S1600536811040220/hb6323Isup3.cml

Additional supplementary materials:  crystallographic information; 3D view; checkCIF report

## Figures and Tables

**Table 1:** Hydrogen-bond geometry (Å, °)

*D*—H⋯*A*	*D*—H	H⋯*A*	*D*⋯*A*	*D*—H⋯*A*
N1—H1⋯O^i^	0.87	2.40	2.9254 (17)	119
N1—H1⋯O^ii^	0.87	2.17	2.8832 (17)	139

Symmetry codes: (i) 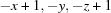; (ii) 
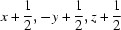
.

**Fig. 1.**
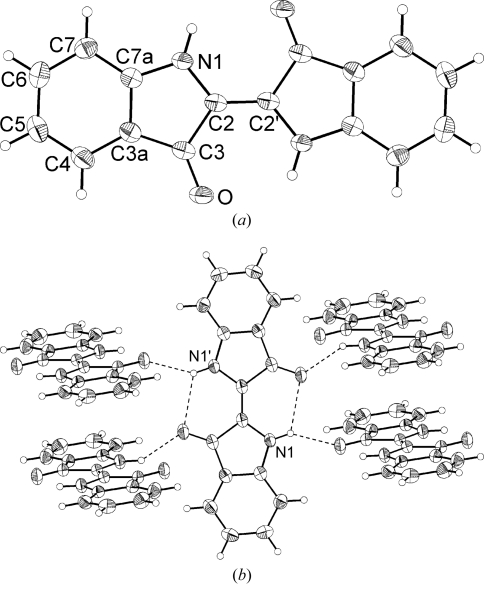
a) Molecular structure of indigo B (50% probability displacement ellipsoids); b) packing diagram of indigo B showing π–π-stacking interactions, inter- (N···O 2.883 (2) Å) and intramolecular (N···O 2.925 (2) Å) hydrogen bonds.

**Fig. 2.**
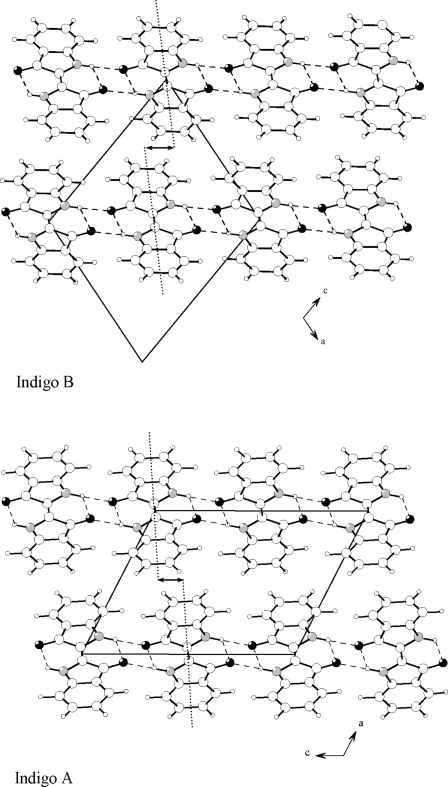
Packing of the indigo layers in indigo B (top) and indigo A (bottom), showing the different offset of the layers; view along [010].

**Fig. 3.**
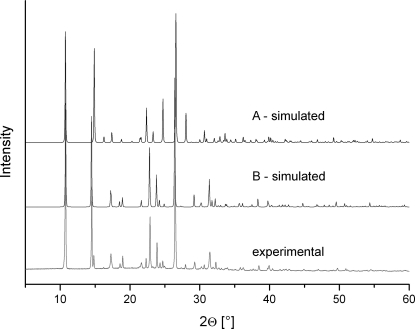
Comparison of the experimental powder diffraction pattern of crushed indigo crystals (bottom, -60 °C, Cu—Kα1) with simulated powder patterns of both indigo modifications, A (top, room temperature, Süsse et al. 1988) and B (middle, -60 °C), based on single-crystal data.

## supplementary crystallographic information

### Comment

Indigo, 2-(1,3-dihydro-3-oxo-2*H*-indol-2-ylidene)-1,2-dihydro-3*H*-indol-3-one, C_16_H_10_N_2_O_2_, is a powerful dye, which is used to colorize for example denim in major industrial processes (Berger & Sicker, 2009). Very important for this application is the chemical stability and the extraordinary low solubility of indigo in water and also in almost all common organic solvents. Even insoluble indigo nanoparticles have been reported recently (Johnson-Buck *et al.* 2009). Von Eller-Pandraud (1958) and Süsse & Wolf (1980) mentioned, that different intermolecular interactions result in two modifications with differences in the packing of indigo molecules. Indigo modification A crystallizes as centrosymmetric molecules in P 2_1_/c with a = 9.24, b = 5.77, c = 12.22 Å, β = 117° and *Z* = 2 (Süsse *et al.*,  1988). Süsse & Wolf (1980) observed indigo modification B as a minor phase with a fraction of about 10% in a mixture with indigo A after sublimation at 10 Torr. We succeeded in preparing single crystals of indigo B as almost pure phase by sublimation at 290 °C at atmospheric pressure. The structure was solved and refined in space group P 2_1_/n, *Z* = 2, with a unit cell setting related to that reported by Süsse & Wolf (1980) (Table 1). Since the quality of the former data set did not allow an interpretation of bond lengths and angles, we used the new crystals to redetermine the crystal structure with improved quality.

The new data set allows a better description of the bonding situation in the indigo molecule. The new values of the bond lengths and angles are consistent with the values reported for indigo A. For example, the bond length between the carbon atoms C2 and its symmetry equivalent C2', previously determined as 1.30 Å in indigo B (1.34 Å in indigo A), which is too short for a delocalized π-system, could be corrected to a distance of 1.359 (2) Å, which is in agreement with a partial double bond. C–C distances within the six-membered ring are in the range of 1.38 to 1.41 Å, consistent with the aromatic character of the ring system.

Responsible for the low solubility of indigo is the intermolecular stabilization by face-to-face π–π-stacking of parallel aromatic rings with a distance of 3.41 Å. Furthermore, each NH and each carbonyl group form strong intermolecular hydrogen bonds N—H···O to one neighbouring unit per functional group with a N···O distance of 2.883 (2) Å (H···O 2.17 Å). In addition, weaker intramolecular hydrogen bonds are observed with N···O 2.925 (2) Å (Figure 1). In both modifications the intermolecular interactions result in almost identical arrangements of indigo molecules in layers, *i.e.* layers parallel (100) in indigo A and layers parallel (-101) in indigo B, respectively. This leads to edge-to-face π-interactions between indigo molecules of adjacent layers. The distance of 3.50 Å for C6 to the neighbouring ring system is in good agreement with literature data (Meyer *et al.*, 2003). Viewing along [010], the difference in the crystal structures of both modifications can be observed as an offset in different directions in the stacking of these layers (Figure 2) (von Eller-Pandraud, 1958). As a consequence, the unit cell volume of modification B is 2.8% larger than that of indigo A, although the data set has been collected at lower temperature (-60 °C).

Powder diffraction data of crushed single crystals of indigo B do not show a phase transition by cooling to -60 °C; the diffraction pattern fits well to the simulated pattern based on our single crystal data. Additional peaks with low intensities are due to small amounts of indigo A (Figure 3).

### Experimental

Crystallization of indigo modification B was achieved by sublimation, similar to the procedure described by von Eller (1955). Indigo was heated to 290 °C in a Schlenk flask at atmospheric pressure. Although indigo is starting to decompose under these conditions, shiny, dark blue, long platelets of indigo B were formed in the cooler region of the glass tube within one hour.

### Crystal data

**Table:**

C_16_H_10_N_2_O_2_	*F*(000) = 272
*M**_r_* = 262.26	*D*_x_ = 1.461 Mg m^−^^3^
Monoclinic, *P*2_1_/*n*	Mo *K*α radiation, λ = 0.71073 Å
Hall symbol: -P 2yn	Cell parameters from 3816 reflections
*a* = 9.799 (2) Å	θ = 1.9–26.0°
*b* = 5.9064 (10) Å	µ = 0.10 mm^−^^1^
*c* = 10.755 (3) Å	*T* = 213 K
β = 106.781 (18)°	Fragment of long platelet, dark blue
*V* = 596.0 (2) Å^3^	1.00 × 0.50 × 0.30 mm
*Z* = 2	

### Data collection

**Table:**

Stoe IPDS 1 diffractometer	896 reflections with *I* > 2σ(*I*)
Radiation source: fine-focus sealed tube	*R*_int_ = 0.033
graphite	θ_max_ = 26.0°, θ_min_ = 1.9°
ω scans	*h* = −12→11
4479 measured reflections	*k* = −6→6
1115 independent reflections	*l* = −13→13

### Refinement

**Table:**

Refinement on *F*^2^	0 restraints
Least-squares matrix: full	H-atom parameters constrained
*R*[*F*^2^ > 2σ(*F*^2^)] = 0.036	*w* = 1/[σ^2^(*F*_o_^2^) + (0.0607*P*)^2^] where *P* = (*F*_o_^2^ + 2*F*_c_^2^)/3
*wR*(*F*^2^) = 0.096	(Δ/σ)_max_ < 0.001
*S* = 1.03	Δρ_max_ = 0.20 e Å^−^^3^
1115 reflections	Δρ_min_ = −0.16 e Å^−^^3^
91 parameters	

### Special details

**Table:**

Geometry. All e.s.d.'s (except the e.s.d. in the dihedral angle between two l.s. planes) are estimated using the full covariance matrix. The cell e.s.d.'s are taken into account individually in the estimation of e.s.d.'s in distances, angles and torsion angles; correlations between e.s.d.'s in cell parameters are only used when they are defined by crystal symmetry. An approximate (isotropic) treatment of cell e.s.d.'s is used for estimating e.s.d.'s involving l.s. planes.

### Fractional atomic coordinates and isotropic or equivalent isotropic displacement parameters (Å^2^)

**Table:**

	*x*	*y*	*z*	*U*_iso_*/*U*_eq_	
N1	0.51536 (12)	0.2222 (2)	0.62685 (11)	0.0300 (3)	
H1	0.5926	0.1963	0.6897	0.036*	
C2	0.46722 (14)	0.0924 (3)	0.51583 (13)	0.0275 (4)	
C3	0.32960 (14)	0.1920 (3)	0.43595 (13)	0.0291 (4)	
C3a	0.30488 (14)	0.3864 (3)	0.50987 (14)	0.0306 (4)	
C4	0.19548 (16)	0.5474 (3)	0.48631 (16)	0.0386 (4)	
H4	0.1176	0.5391	0.4111	0.046*	
C5	0.20430 (18)	0.7188 (3)	0.57588 (17)	0.0419 (4)	
H5	0.1310	0.8269	0.5622	0.050*	
C6	0.32189 (17)	0.7325 (3)	0.68710 (16)	0.0387 (4)	
H6	0.3261	0.8513	0.7463	0.046*	
C7	0.43206 (16)	0.5759 (3)	0.71239 (15)	0.0342 (4)	
H7	0.5108	0.5872	0.7868	0.041*	
C7a	0.42120 (14)	0.4009 (3)	0.62272 (14)	0.0284 (4)	
O	0.25789 (11)	0.1165 (2)	0.32944 (10)	0.0380 (3)	

### Atomic displacement parameters (Å^2^)

**Table:**

	*U*^11^	*U*^22^	*U*^33^	*U*^12^	*U*^13^	*U*^23^
N1	0.0249 (6)	0.0319 (8)	0.0282 (6)	0.0016 (5)	−0.0003 (4)	−0.0006 (5)
C2	0.0234 (6)	0.0297 (10)	0.0270 (7)	−0.0025 (5)	0.0034 (5)	0.0038 (6)
C3	0.0241 (7)	0.0322 (10)	0.0280 (7)	−0.0018 (6)	0.0028 (5)	0.0053 (6)
C3a	0.0279 (7)	0.0317 (10)	0.0311 (8)	0.0006 (6)	0.0068 (6)	0.0041 (6)
C4	0.0327 (7)	0.0423 (11)	0.0382 (8)	0.0080 (7)	0.0064 (6)	0.0058 (7)
C5	0.0413 (9)	0.0378 (12)	0.0489 (10)	0.0121 (7)	0.0164 (7)	0.0053 (7)
C6	0.0440 (9)	0.0322 (11)	0.0447 (9)	−0.0008 (7)	0.0202 (7)	−0.0045 (7)
C7	0.0339 (7)	0.0351 (11)	0.0334 (8)	−0.0048 (6)	0.0091 (6)	−0.0021 (6)
C7a	0.0267 (6)	0.0294 (10)	0.0289 (7)	−0.0014 (6)	0.0079 (5)	0.0044 (6)
O	0.0321 (5)	0.0408 (8)	0.0320 (6)	0.0019 (5)	−0.0051 (4)	−0.0010 (5)

### Geometric parameters (Å, °)

**Table:**

N1—C2	1.3821 (19)	C4—C5	1.383 (3)
N1—C7a	1.394 (2)	C4—H4	0.9400
N1—H1	0.8700	C5—C6	1.403 (3)
C2—C2^i^	1.359 (3)	C5—H5	0.9400
C2—C3	1.4944 (19)	C6—C7	1.387 (2)
C3—O	1.2409 (18)	C6—H6	0.9400
C3—C3a	1.456 (2)	C7—C7a	1.397 (2)
C3a—C4	1.400 (2)	C7—H7	0.9400
C3a—C7a	1.407 (2)		
			
C2—N1—C7a	109.55 (12)	C3a—C4—H4	120.6
C2—N1—H1	125.2	C4—C5—C6	120.45 (15)
C7a—N1—H1	125.2	C4—C5—H5	119.8
C2^i^—C2—N1	126.43 (16)	C6—C5—H5	119.8
C2^i^—C2—C3	125.83 (16)	C7—C6—C5	121.99 (15)
N1—C2—C3	107.74 (13)	C7—C6—H6	119.0
O—C3—C3a	130.39 (13)	C5—C6—H6	119.0
O—C3—C2	124.47 (15)	C6—C7—C7a	117.26 (14)
C3a—C3—C2	105.14 (12)	C6—C7—H7	121.4
C4—C3a—C7a	120.14 (15)	C7a—C7—H7	121.4
C4—C3a—C3	132.53 (14)	N1—C7a—C7	128.36 (13)
C7a—C3a—C3	107.31 (12)	N1—C7a—C3a	110.21 (13)
C5—C4—C3aA	118.71 (14)	C7—C7a—C3a	121.43 (14)
C5—C4—H4	120.6		

Symmetry codes: (i) −*x*+1, −*y*, −*z*+1.

### Hydrogen-bond geometry (Å, °)

**Table:**

*D*—H···*A*	*D*—H	H···*A*	*D*···*A*	*D*—H···*A*
N1—H1···O^i^	0.87	2.40	2.9254 (17)	119
N1—H1···O^ii^	0.87	2.17	2.8832 (17)	139

Symmetry codes: (i) −*x*+1, −*y*, −*z*+1; (ii) *x*+1/2, −*y*+1/2, *z*+1/2.

### Table 2 Relationship between unit cell dimensions of the indigo modifications (Å, °, A^3^)

**Table:**

	Indigo A*^a^*	Indigo B*^b^*	Indigo B*^c^*	Indigo B*^d^*
*a*	9.24 (3)	9.799 (2)	10.84 (1)	9.85 (1)
*b*	5.77 (2)	5.906 (1)	5.887 (6)	5.887 (6)
*c*	12.22 (3)	10.755 (3)	12.28 (1)	10.84 (1)
β	117.0	106.78 (2)	130.02 (5)	107.38 (5)
**a**	580	596.0	600	600
space group	*P* 21/*c*	*P* 21/*n*	*P* 21/*c*	*P* 21/*n*

Notes: (*a*) von Eller (1955); (*b*)  -60°C, this work; (*c*) Süsse & Wolf (1980), original setting; (*d*)  Süsse & Wolf (1980), transformed setting.
